# Current aging research in China

**DOI:** 10.1007/s13238-015-0145-5

**Published:** 2015-03-18

**Authors:** Ruijuan Sun, Heqi Cao, Xudong Zhu, Jun-Ping Liu, Erdan Dong

**Affiliations:** 1 Department of Health Sciences, National Natural Science Foundation of China, Beijing, 100085 China; 2 Institute of Aging Research, School of Medicine, Hangzhou Normal University, Hangzhou, 311121 China

**Keywords:** aging research, funding, objectives and strategies

## Abstract

The mini-review stemmed from a recent meeting on national aging research strategies in China discusses the components and challenges of aging research in China. Highlighted are the major efforts of a number of research teams, funding situations and outstanding examples of recent major research achievements. Finally, authors discuss potential targets and strategies of aging research in China.

## Introduction

According to *China Aging Development Report* (*2013*), the severity of the aging population China faced is rarely seen elsewhere in the world. By the end of 2012, China’s elderly population (aged 60 and above) had totaled 194 million, taking up 14.3% of total population; the elderly population aged 65 and above had reached 123 million—constituting one fifth of the world’s elderly population. By 2030, China’s elderly population is expected to reach 400 million, equivalent to the total population of 15 EU countries. China is the country with not only the world’s largest population, but also a country holds the largest elderly population.

The increases in age-related diseases and the consequent rise in health care costs caused by the decline in the health status along with the aging of the population have brought increasing pressure and burden to the society, families and individuals (Christensen et al., 2009). Therefore, issues such as the rapid increase in the elderly sub-health status, and the prevention and treatment of the aging tissues/organs as well as age-related diseases need to be solved in our aging society. Cell senescence is thought to be the main driving-force of the aging of tissues and organs. The uneven aging of tissues and organs leads to a rapid tissue/organ degeneration, hence resulting in age-related diseases, such as brain degenerative disease, cardiovascular disease, cancer, diabetes, reproductive dysfunction, muscle atrophy, arthritis, and osteoporosis. The solution to cell senescence including promoting stem cell research may provide vital opportunities in tackling of the healthy aging problem, and prevention and treatment of age-related diseases.

Major breakthroughs have been made sometimes in order to understand the mechanisms of how to improve the health issues associated with the aging population. Such challenges are not only faced by China and other developing countries, but are also serious problems for many more economically developed countries. In comparison to other research fields, aging and anti-aging researches have encountered greater magnitudes of challenges, e.g. the inevitable, elusive, and asymptotic biological aging process, the early failure of excessive pursuit of longevity, the deficiency of public understanding in the urgency of aging research in order to preventing aging related diseases. These impediments have hindered aging research development and consequently, led to a dramatic lag behind in preventing aging-related sub-health state and aging-related diseases. Therefore, it is a pressing and significant scientific task to conduct extensive and in-depth studies of the biological process of aging and anti-aging processes in the human body, tissues and organs, to unveil the damage and degeneration caused by aging, as well as the right approaches to enhance and promote anti-aging conditions and mechanisms. Such endeavor will help realize the improvement of the life quality and health of the elderly population, the prevention and early treatment of aging-related diseases, and the relief of the burden on individuals, families and the society originated from the rising aging populations. Currently, clarifying the mechanisms underlying the occurrence and development of aging, gathering anti-aging positive energy to stimulate healthy aging, reducing and postponing the occurrence of geriatric diseases, as well as optimizing life quality and prolonging human lifespan are the popular areas and important goals of the multi-disciplinary (including medicine, life sciences, chemistry, and information sciences) joint research.

## Funding of aging research

**Figure 1:**
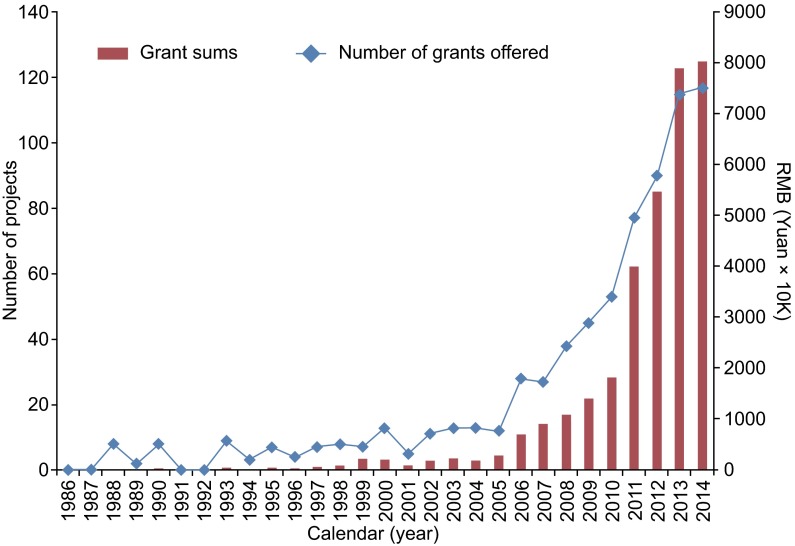
NSFC grant supports to aging research in China since 1986

Research on aging and related diseases in China has received significant support from the National Natural Science Foundation of China (NSFC), the Ministry of Science and Technology of China (MOST), the National Health and Family Planning Commission of China (NHFPC), and local departments over the last decades. NSFC has supported approximately 55 million of US dollars on aging research over the past decade (Fig. 1). The supporting mechanisms vary, including general program, key program, and international joint-program, etc. In the NSFC-CIHR Health Research Cooperation Program in 2009, an aging, geriatric medicine and gerontology research scheme was established separately for the research on body flexibility and aging, cognitive damage and aging. In 2013, the NHFPC and the National Working Commission on Aging jointly issued the China’s Elderly Health Guide aimed at promoting healthy lifestyles, strengthening disease prevention, and improving the life quality and health of the elderly ones. The severity and fast development of population aging, the health care and social insurance problems have become increasingly prominent in China. It has been an important strategic demand and developmental deployment during China’s 12th and 13th Five-Year Plan period. To better promote the health and sustainable development of aging research in China, the Department of Health Sciences, Department of Life Sciences and Policy Bureau of NSFC jointly held the 102th Shuangqing Forum entitled “Key Scientific Issues of Aging Translational Medical Research”, and nearly 50 experts and scholars from more than 30 universities and research institutes at home and abroad attended the forum to discuss national strategies in aging and related diseases. With the aggravation of population aging and the government’s constant seeking of the countermeasures, and for the needs of the society, aging and anti-aging research have become an elevating trend in the society, the academia, science, and technology circle, as well as the service industries. A number of aging-related research laboratories, centers, and research institutions have been established in different cities, sharing research tasks of universities, local governments and the central government. Certain achievements have been made in the aging and age-related disease research, and further development and more breakthroughs on human anti-aging research may be on the way.

## Aging research teams in China

In recent years, China has made significant progress in the research of aging and anti-aging, and the underlying mechanisms. A number of funding programs, such as “Thousand Talents Program (Recruitment Program of Global Experts)”, “National Science Fund for Distinguished Young Scholars”, distinguished professors of “Changjiang Scholars Program”, and “Hundred Talents Program of the Chinese Academy of Sciences” have been in place to encourage biomedical research including aging and anti-aging research. A brief of made-in-China achievements and summaries in distinct aging research fields is summarized as the following.

### Research in organismal, tissue-specific aging, and related diseases

Various lines of research on telomeres in blood vessels, bone marrows, adrenal glands, ovaries were previously reported (Cao et al., 2002; Ju et al., 2007; Bayne et al., 2008; Yang et al., 2009; Bayne et al., 2011). A sharp decline in estrogen levels is associated with an increased impact of oxidative stress on cell telomere length and decreased stem cell activity of follicular granulosa cells during ovarian aging (Wang et al., 2012b; Zuo et al., 2012). Midlife hepatic gene expressions correlated with diet-induced lifespan under different dietary conditions in mice. While increased mitochondrial gene expressions underpin lifespan against aging-related changes, decreased peroxisomal gene expressions extended the lifespan of Drosophila melanogaster and Caenorhabditis elegans by decreasing peroxide levels (Zhou et al., 2012). Mutations of nuclear lamina protein A led to the dislocation of specific histone modification enzyme MOF and SUV39H1 in HGPS and Zmpste24 knockout mice, resulting in epigenetic alterations, chromatin abnormalities and finally impaired DNA repair (Liu et al., 2005; Liu et al., 2012a; Liu et al., 2013). Recently, the mtDNA haplogroup F gene was found to be associated with healthy longevity in female Chuang population, that the allele *CETP 405* *V* is protective in Ashkenazi Jews but may be a risk allele against longevity in the Chinese population, and that the gene interaction between *CETP* and *APOE* polymorphisms confers higher risk of hypertriglyceridemia on Chinese women (Feng et al., 2011; Sun et al., 2013; Sun et al., 2014). In human centenarian samples, Foxo1a, Foxo3a, and β-adrenergic receptor-2 were associated with longevity via regulating vascular function, which may subject to regulation by specific miRNAs to accelerate endothelial cell senescence (Li et al., 2009). Moreover, a new function of Tnni2 in the regulation of bone development may have also advanced current understanding of the pathological mechanism of human DA2B disorder (Zhu et al., 2014).

### Research in the roles of cellular organelles and subcellular structures in aging

Various membranous organelles, including the nucleus, mitochondria, endoplasmic reticulum, peroxisomes, lysosomes, and endosomes, may undergo degenerative changes playing significant roles in cell aging. Current research showed that damages and reduced numbers of cellular organelles (such as mitochondria, endoplasmic reticulum, peroxisomes, and lysosomes) are closely related to the occurrence of aging and aging-related diseases (Wang et al., 2014; Yang and Zhang, 2014; Zhang et al., 2014). Aging and related diseases are also associated with the imbalance of protein homeostasis. Protein stability and normal function in cells are ensured by a precise quality control system including the roles of protein folding, oxidation–reduction of heat shock protein in the endoplasmic reticulum, and the protein degradation system by ubiquitin/non-ubiquitin–proteasome mediated mechanisms (Rubinsztein et al., 2011; Tomaru et al., 2012). The reduction of the mitochondrial respiratory chain transfer efficiency, electronic leak, and ATP synthesis decline often occurred in aging, leading to mitochondrial abnormalities and alteration of reactive oxygen species levels (Green et al., 2011; Vendelbo and Nair, 2011).

A major advance in longevity has been on the metabolic signaling, mitochondrial and autophagy activity regulation. The mTOR inhibitor rapamycin significantly extended the lifespan of laboratory organisms attributed to activation of autophagy (Bjedov et al., 2010; Spong and Bartke, 2012; Wilkinson et al., 2012). Similarly, autophagy inducing agent spermidine has been shown to extend the lifespan of model organisms (Eisenberg et al., 2009). Activating the signaling pathways of EGF increases the number of ubiquitin-proteasomes, and delays the aging of nematodes (Liu et al., 2011b), whilst the lifespan of yeast can be prolonged by deubiquitination enzyme inhibitors to enhance activity of the proteasome (Kruegel et al., 2011). Abnormal mitochondrial function is linked to aging-related diseases. Notably, a newly-cloned receptor of mitochondrial autophagy, FUNDC1, that interacts with LC3 in mediating mitochondrial autophagy, is identified as a target potentially regulating cell aging (Liu et al., 2014). Furthermore, the membrane caveolae protein PTRF is also involved in cell aging (Bai et al., 2011).

In contrast to stress regulation of cellular organelle functions in aging, research in the structural damage in chromosomes has been concentrated on gene loci and such non-coding regions as telomeres. The accumulation of telomere DNA losses including gradually shortened telomere length due to declined telomerase activity plays an important role in the aging process of tissues, organs, and organisms, as well as the development of aging-related diseases (Cong et al., 1999; Cassar et al., 2008; Xu et al., 2008; Nicholls et al., 2012; Ding et al., 2013; Zhou et al., 2014). It has been demonstrated that telomerase mutations cause aging-related diseases such as idiopathic pulmonary fibrosis. Telomerase activity may be closely related to the aging and lifespan of individual organisms, but abnormal activation of telomerase plays a key role in immortalization of cancer cells (Armanios and Blackburn, 2012; Xu et al., 2014). Meanwhile, telomere extension by telomerase requires specific conformational change (Wang et al., 2012a). It has been discovered that telomerase is regulated by protein complexes including telomere protein complex shelterin, a six protein complex of TRF1, TRF2, POT1, TIN2, Rap1, and TPP1, and that TIN2 also plays an important role in mediating the interactions between telomeres and mitochondria (Liu et al., 2004; O’Connor et al., 2006; Xin et al., 2007; Chen et al., 2012; Han et al., 2013; Zhang et al., 2013).

### Research in stem cell aging and regulatory mechanism

The induced pluripotent stem cells (iPSCs) technology enables the creation of cell models of progeria and geriatrics in laboratory conditions, for studying disease mechanisms and drug screening. Meanwhile, human pluripotent stem cells and gene targeting technologies can assist the knockout or modification of any protein from human stem cells, therefore facilitating the functional study of the proteins in organisms with aging and diseases (Liu et al., 2012b). However, the ability of restoring telomere activity by somatic cell nuclear transfer (SCNT) and induced pluripotent stem cells (iPSCs) remains elusive. A recent study demonstrated that embryonic stem cells (Terc^−/−^ ntESCs) from Terc^−/−^ SCNT embryos have stronger differentiation potential and self-renewal capacity compared with that of Terc^−/−^ iPSCs. It is noteworthy that the telomere lengths in SCNT cloned embryos are significantly lengthened, and the capping function of telomeres in Terc^−/−^ ntESCs is enhanced. In addition, the mitochondrial function of Terc/iPSCs and their differentiated derivatives is severely impaired, while the mitochondrial function in Terc^−/−^ ntESCs is considerably improved (Le et al., 2014). Recently, a newly-identified p53 downstream gene, Gadd45a, was found to be activated via p53-dependent and -independent pathways, and involved in the regulation of cellular activity, such as cell cycle checkpoints, apoptosis, DNA damage repair, and signal transduction (Chen et al., 2014). Knockout of miR-142a-3p gene from zebra fish leads to a decline in the number of HSCs in aorta-gonad-kidney (AGM) region, as well as the number of T cells in the thymus. Mechanism study shows that miR-142a-3p regulates the formation and differentiation of HSCs by inhibiting the mediated inflammatory signaling of the interferon regulatory factor 7 (irf7). Of note, miR-142-3p also plays an important role in the formation of HSCs in the AGM region in a mouse, suggesting it plays a highly conserved role in invertebrates (Lu et al., 2013).

### Research in signal transduction and epigenetic regulation of aging

Increasing evidence showed that unlike gene mutation or deletion, epigenetic changes during the aging process of organism can be reversed, making it a promising target to attenuate the aging process. Increases in H4K16 acetylation, H4K20 trimethylation, H3K4 trimethylation, and H3K9 trimethylation levels, and decreases of H3K9 methylation and H3K27 trimethylation levels, have been used as aging-related epigenetic markers (Krishnan et al., 2011; Liu et al., 2012a; Liu et al., 2013). Besides, H3K4 dimethylation accumulates in aging brain in association with two H3K4 methyltransferases, SETD7, and DPY30 (Han et al., 2012), while progressive increases in gene expression and loss of H3K27me3 on IIS components are partly due to increased activity of the H3K27 demethylase UTX-1 during aging (Jin et al., 2011). A recent study revealed that p16 is a downstream gene of FOXA1 and leads to a decline of nucleosome density in aging cell p16 gene promoter region, and thus promoting the transcriptional activation of FOXA1 to p16 (Li et al., 2013). Moreover, p16 3′UTR mRNA methylation provides an important approach for p16 up-regulation in the cell aging process induced by oxidative stress (Zhang et al., 2012b) and that microRNA can identify the coding region and 5′UTR, such as Let-7 that can identify the p66Shc coding region to regulate cellular senescence and lifespan (Xu et al., 2014). Characterized by tissue specificity and high-efficient specificity targeting at target genes, microRNA may be able to provide a new basis for clinical diagnosis and a new therapeutic approach for some age-related diseases.

### Regulation in signaling networks

It is well-known that regulation of insulin-like signaling plays an important role in growth retardation, obesity, type 2 diabetes, life span, and oxidative resistance (Song et al., 2010). The anti-aging effect of YAP2 has been discovered since down-regulating or silencing YAP2 induces replicative senescence of IMR90 human diploid cells in a TEAD and Rb/p16/p53 pathway-dependent manner (Xie et al., 2013). The hepatic IRE1α promotes the adaptive shift of fuel utilization during starvation by stimulating mitochondrial β-oxidation and ketogenesis through the XBP1 s-PPARα axis (Shao et al., 2014). Furthermore, the Grb10/mTOR signaling pathway has been identified to impact the biological changes of adipose tissue and the “brown” process of white adipose tissue (Zhang et al., 2012a; Liu et al., 2014). Intriguingly, RIG-I/MAVS signaling pathway has been found to mediate senescence and aging inflammation. The inflammation response associated with cell senescence is significantly decreased in MAVS-deficient cells and mice. By contrast, interfering with RIG-I in replicative senescence cells can reduce the inflammation response of aging and slow the cell aging process. Further research shows that the anti-aging protein Klotho can act as RIG-I inhibitor to antagonize RIG-I-induced senescence inflammation response, while knockout of Klotho leads to a significantly enhanced inflammatory response (Liu et al., 2011a).

## Significance of basic research on aging

Aging-related diseases are featured by their close relationship to age and they often occur in specific tissues and organs. The aging of specific cell(s) inflames surrounding cells of the same and other cell types leading to tissue degeneration, and continuous accumulation results in irreversible losses of differentiated function and pathological changes of the diseased tissue. Anti-aging is also a process involving corrections and/or removals of damaged molecules, the clearance of senescent cells, as well as proliferation and differentiation of stem/progenitor (precursor) cells. Aging and anti-aging process coexist as aging, anti-aging factors, and injury, repair and regeneration are also in parallel. For instance, interactions of inert and reactive bioactive assemblies and the consequent chemical reaction would free or slowly cross-link the biological molecules, and altered oxidative product may produce damaging molecules precipitating aging of the biological tissues. In addition, aging and damage of cellular organelles may be the pathophysiological basis of many neural aging-related diseases such as Alzheimer’s disease (AD) and Parkinson’s disease (PD), while the replication and regeneration of cells may be the promising approach to vanquish many mitotic aging-related diseases (Liu, 2014). Mobilizing endogenous factors and making use of exogenous anti-aging regimes to repair damages and promote regeneration are important mechanisms to prevent and treat aging-related diseases. Recently, alcohol dehydrogenase 5 (ADH5) is found to be a novel suppressor of neuronal differentiation and maturation, suggesting that ADH5 may improve adult neurogenesis in a physiological or pathological setting (Wu et al., 2014). Aging and anti-aging processes are in mutual checks and balances in both damaging and regenerating. The body condition reaches a steady status through the complicated coordination and interaction of molecules, cells and organs. The imbalances of aging and anti-aging may instigate diseases of related tissues and organs which become the key pathological node for many major diseases occurred simultaneously in elderly people. Despite the inevitability of human aging, aging can be delayed, and many related diseases are preventable.

Aging of the organism, tissues, and organs is the main cause of functional decline, and the premature aging of tissues and organs is the utmost pathological basis for chronic degenerative diseases. Tissue and organ specificity of aging are common for most chronic degenerative diseases, such as AD, atherosclerosis, pulmonary fibrosis, muscular dystrophy, osteoporosis, obesity, diabetes, menopause syndrome, which are related to the premature aging of tissue-specific cells. Aging of organism, tissues, or cells is a complicated and irreversible process, but the time when it occurs and the pace of the process may be regulated. Facing the need to slow down the aging process of the organism and organs, as well as to prevent common chronic diseases, a fundamental understanding of the cause of aging and the causes of aging developing into diseases is a hot topic in the current medical biological research field (Kirkwood, 2008; Lopez-Otin et al., 2013). Currently, extensive research has been laid on the homology of the pathogenic causes for many aging-related diseases and the fundamental mechanism for organism aging. Research on aging-inducing genes or the aging genes, their mechanisms and unknown aging-related genes has been attracting increasingly more attention (Hayflick, 2007; Wolff and Dillin, 2013). Multiple causes for aging-related diseases of organism include: (1) Chromosomal damages, gene mutations, and epigenetic abnormalities; (2) Mitochondrial damages, accumulation of lipid peroxidation products and lipofuscin caused by reactive oxygen species; (3) Diabetes caused by high blood sugar and accumulation of advanced glycation end products in a variety of cells and tissues; (4) Denatured fracture and cross-linking of collagen and elastin; (5) Protein misfolding, denaturation, and accumulation of insoluble polymer; (6) Abnormal transport of small molecules, recycling barriers of cells, organelles, proteins, and other macromolecules; (7) Tissue inflammation, cell interactions, and homeostasis disorders; (8) Homeostasis decline caused by imbalance of hormone and immune cells etc. (Green et al., 2011; Blagosklonny, 2012). All these problems are likely to cause lesions of specific organs and systems, resulting in aging-related diseases.

Challenges and weaknesses in aging research in China remain obvious in manifolds. Most importantly, aging (especially premature aging) has not been well recognized as the primary factors of most non-infectious chronic diseases. Although the central government has been increasing its research budgets continuously in aging research, contributions from the state governments and the private sections have not been considered as high priority to aging research and development. As a matter of fact, with improved living conditions and life expectancies in China, there are significant demands and interests of the general population in detecting premature aging and delaying aging occurrence. In addition, international collaborations in promoting aging research have until recently been on the agenda including our organizing the “Shuangqing” forum and a number of other activities from universities, hospitals, and Chinese Academy of Science. Unfortunately, a national-wide database incorporating information of aging-related resources is yet to be established, which requires urgent actions from aging research organizations and researchers. On the other hand, collaborations between hospitals and basic research institutes are lacking which hinders the development of gerontology and geriatrics. The phenomenon of being more difficult for an aging disease to find its way to geriatrics than for ill children to be sent to pediatrics may well reflect currently poor understandings of the mechanisms and biomarkers of aging bodies for clinical practice, making the translational aging research struggle to develop. While many challenges and weakness of aging research in China may be shared by global situations, we highlight at least but not last the following a few that are particularly sought after for development in China:

(1) The impact of particular environmental factors, various foods, and exercise levels on aging and lifespan, (2) Genetic traits and epigenetic specificities affected by stress in premature aging and aging related diseases including metabolic disorder and cancers in liver, digestive tracts, and lung, (3) Effect of endocrine/paracrine/autocrine hormones and cytokines, microorganisms, inflammatory/immune responses on tissue and organ aging, (4) Repair mechanisms of stem cells and key enzyme molecules in aging damages, (5) Tissue- and organ-specific mechanisms of aging and diagnostic markers.

## Objectives and strategies of aging research

Numerous tasks need to be done prior to achieving the final goal of preventing and reducing aging-related diseases. By means of original innovative research, it would be important to achieve 3 major comprehensive objectives. Firstly, at the molecular and cellular levels, it would be important to understand the basis of aging, to clarify the molecular characteristics of different types of cellular aging and their roles in the aging of tissues and organs, regulatory networks, plasticity, and clearance mechanisms; to clarify the regulatory mechanism of the change of sub-cellular structure of eukaryotic cell aging in aging and anti-aging process; to clarify the basic structure and spatial conformation of different key molecules and to analyze the binding structural domain of interacting molecules and small molecules; to analyze direct and indirect (epi-)genetic regulatory network of different genes, especially to identify and control the key transcription factors mediating aging process; to unveil the regulation and effect of key environmental factors, especially the microflora in different tissues (such as intestine), excessive nutrients, toxic molecules, as well as exercises and sleep, etc. on the aging of tissues and organs.

Second, at the levels of tissue- and organ-specific aging, it would be important to clarify the transition of aging damage to pathological damage and the composition and structural alteration; to clarify the source, function, and regulatory environment of triggers to aging and aging-related diseases; to clarify the susceptible parts of aging organelle/tissue/organ, and the complicated etiology of aging-related diseases; to rationalize and establish the relationship among the causes, to strengthen the research in the compensatory effect of aging development into aging-related diseases; to set out the mechanism, reagents, and methods for early intervention in the transition from aging to aging-related diseases in the micro environment; to clarify the definition of physiological aging and pathological aging of organisms as well as the definition of healthy lifespan; to clarify the similarities and differences in the lifespan regulation of non-vertebrates, invertebrates, and mammals and to clarify the disease susceptibility and resistance mechanisms of different models to aging-related diseases; to clarify the functional ingredients of natural medicine (including Chinese medicine), the role of new, small molecule compounds and clinical drugs (such as rapamycin, metformin), and the regulatory mechanism of other environmental factors to mammalian lifespan.

Third, at the levels of particular diseases, it would be important to clarify the role of signaling pathway impairment and pathological basis in the occurrence and development of aging-related diseases through the analysis of animal/human disease models; to clarify the role of stem (precursor) cells and their aging in aging and different common aging-related diseases; to clarify the molecular biological characteristics and development in the research models of brain aging-related diseases (such as PD, AD and common cognitive impairment), vascular aging-related diseases, marrow aging-related diseases, lung aging-related diseases, and ovarian aging-related diseases; to clarify the mechanisms of prevention of aging-related diseases via either inhibiting key aging molecules or activating anti-aging molecules, thus providing the basis and strategies for further research on the prevention and treatment.

It is possible that senescence of a single or a group of cells in certain tissues and organs seeds tissue specific aging which paves, and is a prelude of, aging-related diseases. Such senescent cells suffer irreversible damages, therefore being amplified to cause loss of normal function of aged tissues and organs. Current aging research in China may need to collaborate with researchers in other countries to integrate the approaches of molecular imaging, stem cell biology, molecular genetics and genomics, molecular pathology, histology and embryology, systematic biology, bioinformatics, and clinical medicine to conduct research at different levels of aging and related diseases. Research of aging and aging-related diseases is a great challenge and opportunity in the 21st century. Although it has experienced a long history, aging research is still limited especially on the transition from aging to aging diseases. In-depth and extensive exploration on key molecules and critical mechanisms are still very much sought after. The complexities of various body tissues and differences in structures and functions bring massive difficulties in human anti-aging research. However, the discovery of stem cell plasticity, and continuous breakthroughs in verification of the causal relationship between molecules and aging-related diseases, for example, using different cell culture systems and animal models, have provided opportunity for collaborative innovation in the research on aging and aging-related diseases.
